# Supplementary material for: Identification of Three Novel Linear B-Cell Epitopes in Non-Structural Protein 3 of Porcine Epidemic Diarrhea Virus Using Monoclonal Antibodies
Source: Viruses. 2024 Mar 9;16(3):424. doi: 10.3390/v16030424 (PMC10975687; doi:10.3390/v16030424)
Supplement: Supplementary file 1 [file viruses-16-00424-s001.zip › viruses-2876681-supplementary/Supplementary Materials.pptx]

## Slide 1
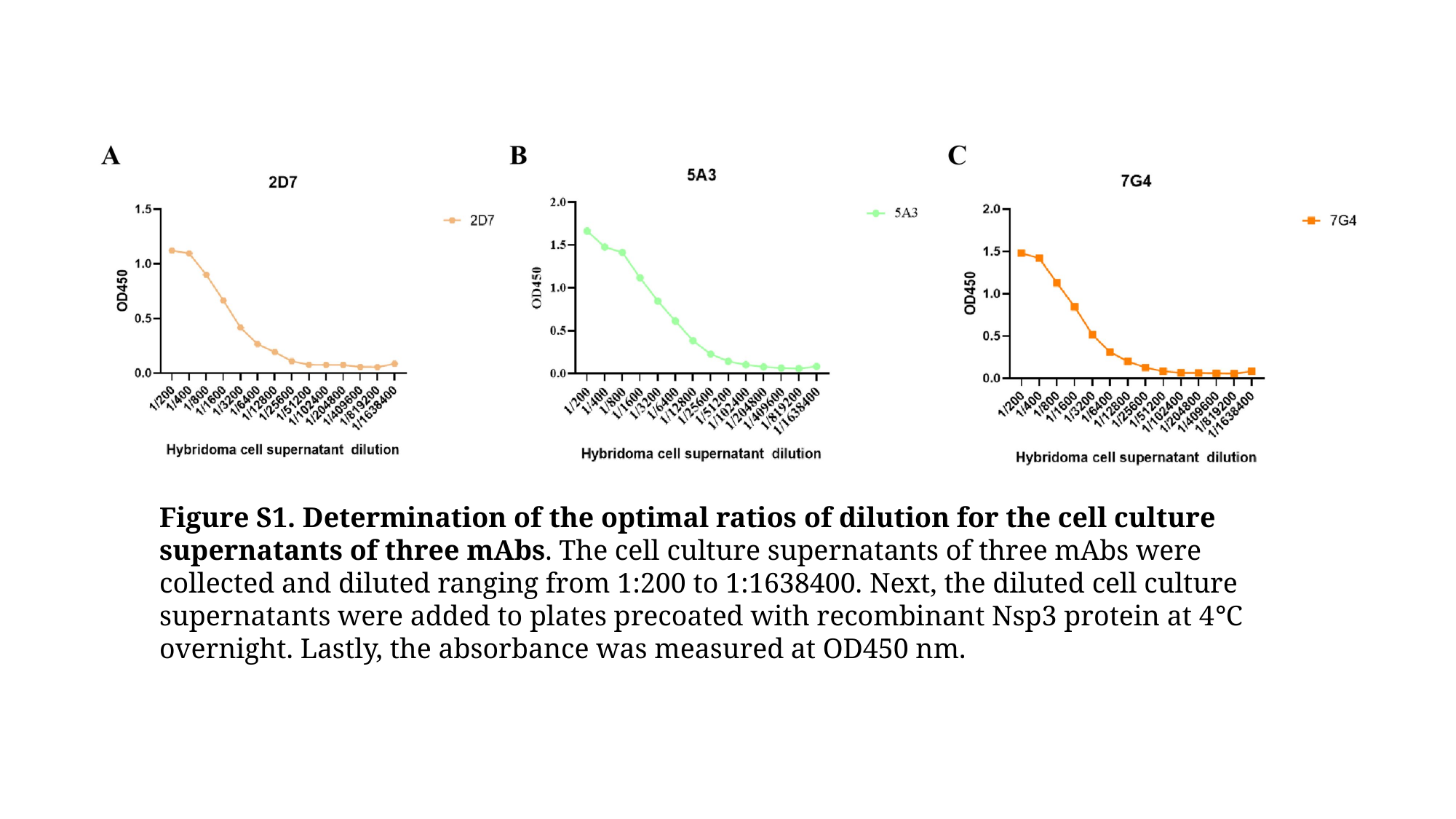

Figure S1. Determination of the optimal ratios of dilution for the cell culture supernatants of three mAbs. The cell culture supernatants of three mAbs were collected and diluted ranging from 1:200 to 1:1638400. Next, the diluted cell culture supernatants were added to plates precoated with recombinant Nsp3 protein at 4℃ overnight. Lastly, the absorbance was measured at OD450 nm.
